# Acute Gallstone Pancreatitis: If a Picture Is Worth a Thousand Words, How Many Images Do We Need?

**DOI:** 10.7759/cureus.33666

**Published:** 2023-01-11

**Authors:** Si E Chen, Qamar Iqbal, Sreelakshmi Mallappa

**Affiliations:** 1 General Surgery, The Hillingdon Hospitals NHS Foundation Trust, Uxbridge, GBR; 2 General and Colorectal Surgery, The Hillingdon Hospitals NHS Foundation Trust, Uxbridge, GBR

**Keywords:** choledocholithiasis, computed tomography (ct ), ultrasound, magnetic resonance cholangiopancreatography (mrcp), gallstone pancreatitis

## Abstract

Introduction

Accurate diagnosis and prompt definitive management of choledocholithiasis are vital in acute gallstone pancreatitis. The sensitivity of detection of choledocholithiasis varies across imaging modalities. Magnetic resonance cholangiopancreatography (MRCP) is the most sensitive but may not be necessary, resulting in both delayed definitive management and increased costs. We aimed to evaluate the range of radiological investigations patients with acute gallstone pancreatitis underwent and the clinical appropriateness of MRCP when performed.

Methods

This was an observational study of patients diagnosed with acute gallstone pancreatitis between January 1, 2019 and November 30, 2021 in a district general hospital in London, UK. A detailed review of patient records, laboratory and radiological results, and endoscopic and/or operative intervention was undertaken.

Results

One hundred consecutive patients diagnosed with acute gallstone pancreatitis (median age 57 years) were included. Seventy-nine had a transabdominal ultrasound (USS), 46 had CT, and 40 patients had MRCP. The median waiting time for these investigations was 1, 0, and 4 days, respectively. Choledocholithiasis was identified in 21 patients (4 on USS, 5 on CT, and 12 on MRCP). As definitive management, 37% underwent endoscopic retrograde cholangiopancreatography, and 57% underwent laparoscopic cholecystectomy. A total of 19% of patients were readmitted with pancreatitis prior to definitive management.

Conclusions

First-line imaging investigations such as USS and CT can detect some cases of choledocholithiasis in patients with acute gallstone pancreatitis, but not all. Despite expenses in terms of cost and length of hospital stay, MRCP remains an essential resource to detect cases of choledocholithiasis not captured by USS or CT. We recommend establishing a guideline to streamline imaging in assessing acute gallstone pancreatitis.

## Introduction

The etiology of acute pancreatitis can be accounted for by gallstones in 40-60% of cases [1]. The pathophysiology of acute gallstone pancreatitis can be attributed to obstruction of the common bile duct (CBD) by migrating gallstones [2,3]. More than 70% will spontaneously pass the obstructing stone into the duodenum, but 3-7% will develop persistent obstruction, leading to acute pancreatitis [4,5]. In such patients, prompt identification and management reduce the incidence of severe pancreatitis, which can otherwise lead to morbid sequelae such as fluid imbalance and sepsis, with mortality of up to 9% and a 29%-67% risk of recurrent pancreatitis [6-8]. Definitive management strategies include laparoscopic cholecystectomy and/or endoscopic retrograde pancreatography (ERCP) to manage choledocholithiasis [9].

Imaging modalities used to detect choledocholithiasis in patients with acute pancreatitis often include transabdominal ultrasound (USS), CT, magnetic resonance cholangiopancreatography (MRCP), endoscopic ultrasound (EUS), and endoscopic retrograde cholangiopancreatography [10]. These vary in terms of sensitivity and accuracy, radiation exposure and invasiveness, ease of access/expertise, waiting time delays, and cost [10-12]. Invasive methods such as EUS and ERCP are the designated gold standard for detecting choledocholithiasis but are associated with higher costs and risks associated with the procedure and are rarely routinely available at smaller district general hospitals [6]. Non-invasive imaging methods such as USS and CT are low cost but have lower sensitivities in the detection of biliary tract stones ranging from 22% to 55% for USS and 25% to 90% for CT [13,14]. MRCP, another non-invasive imaging technique, has high sensitivities for detecting choledocholithiasis in acute gallstone pancreatitis of 77-100% [6,10] but is conversely associated with higher cost and delays in patient stay due to limited availability [15].

First-line diagnostic tests typically include liver function tests (LFTs) and ultrasonography [10], which can provide sufficient information to make a definitive diagnosis preceding therapeutic management without MRCP or any other imaging investigations [13]. The National Institute for Health and Care Excellence (NICE) guidelines currently recommend MRCP only if prior imaging has detected CBD dilatation but not choledocholithiasis and/or if the LFTs are abnormal and recommends that the patient receives definitive management within two weeks of initial presentation with gallstone pancreatitis [9,16]. If MRCP is performed when not clinically indicated, this could result in increased length of stay, patient anxiety, and ultimately increased cost expenses for the NHS [17]. In our study, we aimed to evaluate the number and type of radiological investigations patients with acute gallstone pancreatitis underwent at our Trust and the clinical appropriateness of MRCP if performed.

## Materials and methods

This was a retrospective observational study of patients diagnosed with gallstone pancreatitis between January 1, 2019 and November 30, 2021. The study setting was a district general hospital on the outskirts of London, UK. Diagnosis of acute pancreatitis was defined as amylase levels greater than three times the upper limit of the normal range for our laboratory values and/or imaging evidence of acute pancreatitis. Gallstone pancreatitis was diagnosed by the presence of gallstones on imaging undertaken on this or prior admissions. A detailed review of patient records, laboratory and radiological results, and endoscopic and/or operative intervention was undertaken.

Transabdominal USS was performed and reported by professional sonographers, while CT and MRCP investigations were reported by consultant radiologists. CBD dilatation was defined as CBD diameter ≥7mm. Choledocholithiasis was recorded as present if noted in imaging reports or absent if not. Several imaging reports did not mention either the presence or absence of choledocholithiasis, and we have classified these as ‘not applicable’ (N/A). The decision to proceed to MRCP or ERCP was a clinical consultant-led decision, considering imaging reports and LFT results.

In all patients who had ERCP (37%), biliary sphincterotomy and balloon trawl of the common bile duct were performed to identify and retrieve any CBD stones. Successful ERCP was defined as the retrieval of stones or debris from the common bile duct. Readmission was defined as patients who were represented to the hospital with acute gallstone pancreatitis on a subsequent, discrete admission prior to the instigation of definitive management. Cost estimations were obtained from NHS England National Tariffs 2021-2022 [18].

## Results

Overall, 100 patients were diagnosed with acute gallstone pancreatitis between January 2019 and November 2021. The median age was 57 (range 13-100), and 71% were female. A total of 56% had abnormally raised bilirubin, 77% raised alanine transaminase (ALT), and 56% raised alkaline phosphatase (ALP) on admission.

A total of 79% had USS, 46% had CT, and 40% had MRCP. A total of 29/40 (72.5%) had inpatient MRCP, and 11/40 (27.5%) had outpatient MRCP post-discharge. The mean number of imaging investigations per patient was 1.66 (range 1-3). Median waiting time from admission to the investigation was 1 (range 0-10), 0 (range 0-8), 3 (range 1-6), and 14 (range 8-69) days for USS, CT, inpatient MRCP, and outpatient MRCP respectively. A summary of the results is provided in Table 1.

**Table 1:** Summary of findings of CT, USS and MRCP. USS: Ultrasound; MRCP: Magnetic resonance cholangiopancreatography.

	Common bile duct (CBD) not evaluated	Gallstones seen	Bile duct dilated	Choledocholithiasis	Total number of patients who had investigation	Median waiting time (days)
USS	8	68	24	4	79	1 (range 0-10)
CT	11	17	17	5	46	0 (range 0-8)
Inpatient MRCP	0	24	10	9	29	3 (range 1-6)
Outpatient MRCP	0	10	3	3	11	14 (range 8-69)

Transabdominal ultrasound (USS)

**Figure 1:**
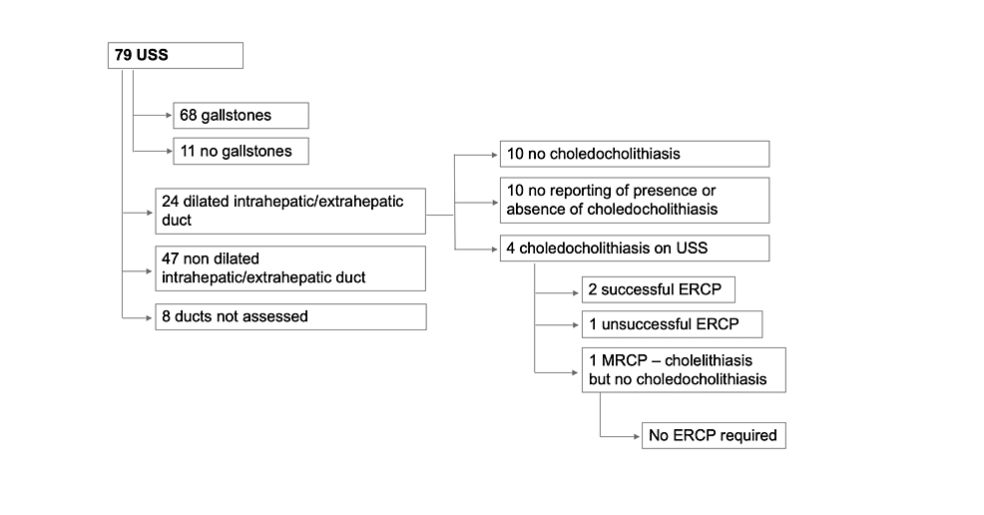
Flow chart showing findings and outcomes in patients who had transabdominal ultrasound (USS).

Of the 79 patients who had USS, 68/79 (86%) had gallstones identified, and 24/79 (30%) had the presence of dilated intrahepatic or extrahepatic bile ducts (Figure 1). A total of 4/79 (5%) patients were diagnosed with choledocholithiasis on USS. However, one of these four (25%) later had MRCP, which showed no evidence of choledocholithiasis. Thirty (38%) patients who had USS had subsequent MRCP, and an additional 11/30 (37%) of these patients were found to have choledocholithiasis on MRCP. All 11 patients were offered ERCP with biliary sphincterotomy and balloon trawl, but one declined, and one had ERCP at a tertiary center. Eight of the nine patients who had ERCP (89%) had CBD stones or debris removed on balloon trawl during ERCP.

Computed tomography (CT)

**Figure 2:**
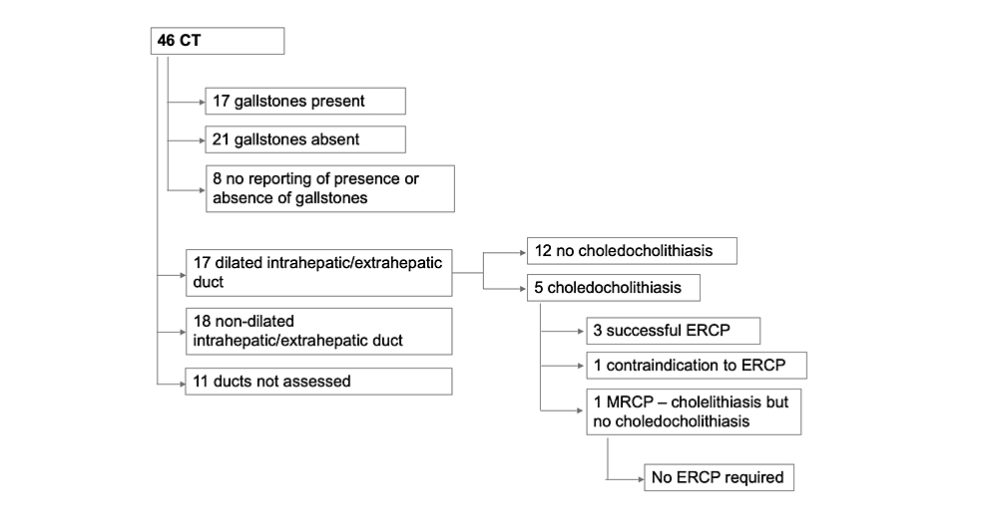
Flow chart showing findings and outcomes in patients who had CT.

Of the 46 patients who had CT, 17/46 (37%) had gallstones identified, and 17/46 (37%) had the presence of dilated intrahepatic or extrahepatic bile ducts (Figure 2). A total of 5/46 (11%) patients were diagnosed with choledocholithiasis on CT; however, one of these five (20%) later had MRCP, which did not show any evidence of choledocholithiasis. A total of 17 patients who had CT had subsequent MRCP (37%), and an additional 4/17 (23.5%) patients were found to have choledocholithiasis on MRCP, all of whom had subsequent ERCP with balloon trawl and sphincterotomy. Two of these four patients had stones removed on balloon trawl; one had an unsuccessful ERCP; and one did not have a dilated CBD on ERCP, nor were any stones or debris retrieved.

Magnetic resonance cholangiopancreatography (MRCP)

**Figure 3:**
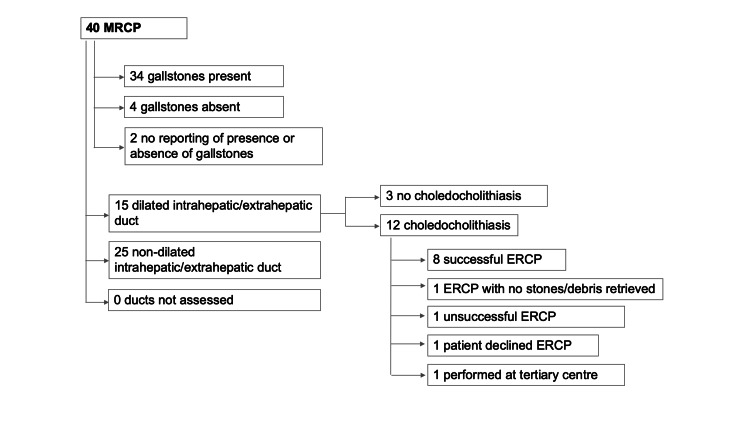
Flow chart showing findings and outcomes in patients who had MRCP. MRCP: Magnetic resonance cholangiopancreatography.

Overall, 40 MRCPs were performed in our cohort; 29 as inpatients and 11 as an outpatient. A total of 34/40 (85%) had gallstones visualized (Figure 3). A total of 15/40 (37.5%) had dilated CBD: 12/15 (80%) had inpatient MRCP, and 3/15 (20%) had outpatient MRCP. A total of 12/40 (30%) patients had choledocholithiasis identified on MRCP; four did not have any evidence of dilated ducts as obstructing stones were <7mm in size. Three of four patients had prior USS, of which two patients did not have duct dilatation visualized on USS, and in one patient, CBD was not clearly visualized on USS. Hence, no comment could be made on the presence or absence of duct dilatation.

All 12 patients with choledocholithiasis were offered ERCP. One had ERCP at a tertiary center, and one declined ERCP. Eight patients had successful ERCP with stones or debris removed, one had ERCP with no stones or debris found, and one had unsuccessful ERCP wherein the CBD could not be cannulated.

A total of 77% of patients either had dilated CBD on prior imaging or the presence of deranged LFTs and thus had clinically indicated a need for MRCP in accordance with NICE guidelines [9]. A total of 33% of patients who warranted MRCP received one; 44% of patients who should have had MRCP for dilated CBD or deranged LFTs did not. This was for various reasons, including improvement in LFTs, contraindications to MRI, proceeding straight to ERCP, or other clinical considerations. Subsequent MRCP was not warranted in 23% of patients, as 12% did not have dilated CBD or deranged LFTs, 3% did not have deranged LFTs, but radiology reports did not describe the diameter of CBD, and 8% had confirmed choledocholithiasis on prior CT or USS imaging. Six MRCPs were performed in patients with no clinical indication for MRCP as per NICE guidelines; two patients had had prior choledocholithiasis seen on either CT or USS, but neither had choledocholithiasis confirmed on MRCP. Two patients with MRCP in an outpatient setting did not have deranged LFTs or duct dilatation seen on USS. An additional two patients did not have deranged LFTs and the CT or USS did not report the presence of CBD dilatation or choledocholithiasis.

Outcomes

As definitive management, 37% of patients had ERCP. Twenty-two had inpatient ERCP during the initial hospital stay for acute pancreatitis, nine had outpatient ERCP post-hospital discharge, and six had ERCP on a subsequent admission. Median waiting time was 4.5 days for inpatient ERCP (range 0-15) and 13 days for outpatient ERCP (range 9-111). A total of 18/37 (49%) had CBD stones retrieved on balloon trawl on ERCP: 10/18 were inpatient ERCP, 6/18 were outpatient ERCP, and 2/18 were ERCP performed on subsequent admission. A total of 5/37 (13.5%) had debris removed. A total of 11/37 (30%) had no choledocholithiasis identified, 2/37 (5.5%) had unsuccessful duct cannulation, and 1/37 (2%) had ERCP performed at a tertiary center.

A total of 54/100 patients had a laparoscopic cholecystectomy, and 8/100 are currently on the waiting list as of March 1, 2022, with a median waiting time of 66 days (range 0-350 days). Median waiting time prior to the SARS-CoV-2 pandemic was 51 days (range 0-298) and 99 days (range 0-350) during the pandemic.

Twenty-two patients had ERCP followed by laparoscopic cholecystectomy. Seven patients had a previous laparoscopic cholecystectomy before this hospital admission for pancreatitis. Eight patients were transferred to other hospitals for further management and lost to follow-up. Two patients were not fit for intervention, given other co-morbidities, and five had no definitive management. 

The median length of stay for patients who did not have MRCP was four days (range 1-36), compared to five days (range 1-27) for patients who had MRCP. A total of 19% of patients were readmitted with a recurrent episode of gallstone pancreatitis while waiting for their definitive management. Mortality was 5%. Causes of death included viral pneumonitis, cerebrovascular accident, pancreatic carcinoma, and frailty combined with acute gallstone pancreatitis. The cause of death could not be elucidated in two patients as they passed away outside of our hospital.

## Discussion

Several studies have been conducted on the accuracy and sensitivity of MRCP in evaluating choledocholithiasis in acute gallstone pancreatitis [6,10]. However, there has so far been limited data evaluating the necessity of MRCP in acute gallstone pancreatitis and its cost implications for our healthcare system. Our study is novel in its attempt to assess this in a resource-limited district general hospital setting. We aimed to evaluate the number and type of imaging investigations performed on patients admitted with acute gallstone pancreatitis to determine whether MRCP was necessary before proceeding to definitive management. There have been several studies to date advocating the use of MRCP in diagnosing choledocholithiasis prior to ERCP [19,20]. Foremost, the detection of biliary dilatation by ultrasonography has a poor sensitivity of 55% [21]; 10% choledocholithiasis would be missed by USS and deranged LFTs alone without more detailed imaging such as MRI or endoscopic USS performed [19]. MRCP is highly accurate in the detection of CBD stones, with a recent meta-analysis reporting a summary sensitivity of 0.93 (95% CI: 0.87-0.96) and specificity of 0.96 (95% CI: 0.9-0.98) [6,10]. With high sensitivity and specificity, combined with its non-invasiveness and lack of radiation, MRCP is a beneficial investigation at one's disposal. However, given cost and resource limitations in the healthcare system, particularly in district general hospitals, rationing its use with appropriate patient selection is key.

In our study, 78% of patients with choledocholithiasis identified on initial CT or USS proceeded directly to ERCP: on balloon trawl, 50% had stones retrieved, and 50% had debris retrieved. First-line diagnostic tests, including LFTs and ultrasonography, can therefore provide sufficient information to make a definitive diagnosis, allowing patients to be streamlined directly to ERCP or other definitive management strategies [10,13]. Per the NICE guidelines, MRCP is only required if prior imaging detected CBD dilatation but not choledocholithiasis and abnormal LFTs [9].

This relies on detailed reporting of the appearance of the CBD. In our study, eight USS and 11 CT imaging reports did not discuss CBD dilatation or the presence of choledocholithiasis. Two of these patients underwent unnecessary outpatient MRCP, which did not show biliary duct dilatation or choledocholithiasis. More detailed initial radiology reporting providing necessary details on CBD diameter and/or presence of choledocholithiasis is therefore of the utmost importance to reduce unnecessary MRCP investigations. However, in our study, 41.6% who had choledocholithiasis identified on MRCP had stones <7mm; four of these patients (80%) had stones retrieved on ERCP. These were not detected on earlier USS or CT imaging in any of the five patients. Smaller stones situated in the CBD may not cause duct distension; ergo, such cases of choledocholithiasis are more likely to be missed by USS and CT and require more sensitive imaging modalities such as MRI.

MRCP is more specific when performed later, as this allows time for smaller stones to pass spontaneously. Cavdar F et al. recommend delaying MRCP for up to a week after the initial presentation if clinically stable to maximize specificity [22]. In our study, two patients with prior choledocholithiasis visualized on initial USS and CT imaging underwent MRCP. Although MRCP was not indicated, CBD stones were not identified on MRCP, preventing an unnecessary ERCP from being performed. It is likely the stones had passed prior to MRCP as these patients had a four and five-day wait, respectively. However, the long waiting time for MRCP may delay definitive management. The median waiting time for MRCP was 4.5 days in our study compared to other hospitals [23]. In our hospital, ERCP lists run twice weekly, with a median waiting time of eight days. If MRCP was performed prior to every ERCP, this would add a significant number of days to inpatient care, with costs of £277 per day of inpatient stay [18].

MRCP is furthermore itself an expensive investigation. According to the NHS England National Tariff 2022/2023 [18], an MRCP costs £114 to perform and £23 to report. A CT scan costs £55 with contrast and £21 to report. Ultrasonography is the cheapest option, costing £43 in total to perform and report. Coupled with an increased duration of admission of one day in patients who had MRCP in our study, this further adds to hospital costs and exacerbates bed pressures. A recent study by Ward WH et al. reports that MRCP did not result in increased length of stay [20]. However, this is likely due to more readily available MRI machines in larger centers where this study was undertaken and may not translate to district general hospital settings where such resources are limited. The median waiting time for laparoscopic cholecystectomy in our study was 66 days, thereby not meeting recommendations to perform surgery within two weeks [16]. A considerable increase in waiting time of 48 days was noted due to both waves of the SARS-CoV-2 pandemic. This was coupled with a considerable delay in restarting a well-established 'hot laparoscopic cholecystectomy' pathway within our Trust as theatre space and personnel in the post-pandemic recovery phase had to be prioritized to meet cancer targets. Patients unwilling to have surgery, on-the-day cancellations due to emergency cases, and lack of theatre space added to lengthy delays during the post-pandemic recovery phase. This is now being addressed urgently in an active attempt to meet NICE recommendations for managing gallstone pancreatitis.

Limitations of our study

The scope of our study was limited by the small sample size in a single center, district general hospital experience. Due to resource limitations, there may be an exaggeration of the waiting time for MRCP as compared to tertiary centers, contributing to increased length of inpatient stay. However, this also reflects and accentuates the reality of poor accessibility of MRCP in a district general hospital setting and highlights the need to streamline imaging investigations in acute gallstone pancreatitis for optimal patient care. The two waves of the COVID pandemic during the timeframe of our study also contributed to delays in definitive management. Unfortunately, we were unable to calculate sensitivity or specificity as this was a retrospective study and patients had ERCP according to clinical need only, so the total number of true negative results could not be determined.

There may be bias in determining which patients proceeded to MRCP or ERCP. Although based on laboratory results and imaging, this was a consultant-led decision based ultimately on the clinical picture. As a result, NICE guidelines were not always strictly adhered to; some were more inclined to request MRCP even when not indicated according to NICE guidelines and vice versa.

Furthermore, mobile gallstones in the bile duct seen on USS or CT may have spontaneously passed by the time of MRCP or ERCP due to waiting time delays, which may have contributed to the high false positivity rate of these investigations. 

Future directions

Alternative methods to determine whether patients should undergo MRCP imaging would be to develop a scoring system to assess the likelihood of choledocholithiasis. A recent pediatric model involving ALT, ALP, total bilirubin, and CBD diameter on USS has been developed, yielding an overall accuracy of 71.5% in the detection of choledocholithiasis [24]. Sherman JL et al. proposed a scoring system based on the degree of elevation of LFTs combined with CBD diameter [25]; this could be explored in conjunction with newer methods utilizing artificial intelligence [26]. Other diagnostic techniques, such as intraoperative cholangiography, may also negate the need for MRCP in patients undergoing laparoscopic cholecystectomy for acute gallstone pancreatitis [27]. Future randomized controlled trials on cost evaluations of MRCP may also provide further insight into the necessity and utility of MRCP in symptomatic gallstone disease in the NHS system [28]. A start to improve services provided would be to have a multidisciplinary team approach to patient care, involving the radiology department personnel in decision-making regarding further investigations and reiterating the importance of detailed radiology reporting.

## Conclusions

Although first-line imaging tests such as USS and CT can provide sufficient information to form a definitive diagnosis of choledocholithiasis in certain patients with acute gallstone pancreatitis, our results show that these are by no means infallible investigations, as several cases of choledocholithiasis remained undetected in our cohort. This is further hindered by the lack of a detailed description of the CBD in some of these initial USS and CT radiology reports. MRCP is a more sensitive imaging modality, particularly for smaller stones in CBD, and therefore, remains an essential secondary line imaging resource. However, MRCP is not without disadvantages, notably the longer length of stay, cost, and in some cases, delayed definitive management via ERCP or laparoscopic cholecystectomy. We recommend conducting a formal cost-benefit analysis of MRCP in patients with acute gallstone pancreatitis and establishing a guideline in conjunction with radiology to streamline imaging investigations and management options.
